# A positive fluid balance is associated with a worse outcome in patients with acute renal failure

**DOI:** 10.1186/cc6916

**Published:** 2008-06-04

**Authors:** Didier Payen, Anne Cornélie de Pont, Yasser Sakr, Claudia Spies, Konrad Reinhart, Jean Louis Vincent, the Sepsis Occurrence in Acutely Ill Patients (SOAP) Investigators

**Affiliations:** 1 Department of Anesthesiology and Intensive Care, CHU Lariboisière, 2, rue Ambroise – Paré, F-75475 Paris Cedex 10, France; 2 Adult Intensive Care Unit C3-327, Academic Medical Center, University of Amsterdam, Meibergdreef 9, NL-1105 AZ Amsterdam, The Netherlands; 3 Department of Anesthesiology and Intensive Care, Friedrich-Schiller-University Jena, Erlanger Allee 101, D-07747 Jena, Germany; 4 Department of Anaesthesiology and Intensive Care, Charité-Universitätsmedizin Berlin, Hindenburgdamm 30, D-12200 Berlin, Germany; 5 Department of Intensive Care, Erasme Hospital, Université libre de Bruxelles, 808, Route de Lennik, B-1070-Brussels, Belgium

## Abstract

**Introduction:**

Despite significant improvements in intensive care medicine, the prognosis of acute renal failure (ARF) remains poor, with mortality ranging from 40% to 65%. The aim of the present observational study was to analyze the influence of patient characteristics and fluid balance on the outcome of ARF in intensive care unit (ICU) patients.

**Methods:**

The data were extracted from the Sepsis Occurrence in Acutely Ill Patients (SOAP) study, a multicenter observational cohort study to which 198 ICUs from 24 European countries contributed. All adult patients admitted to a participating ICU between 1 and 15 May 2002, except those admitted for uncomplicated postoperative surveillance, were eligible for the study. For the purposes of this substudy, patients were divided into two groups according to whether they had ARF. The groups were compared with respect to patient characteristics, fluid balance, and outcome.

**Results:**

Of the 3,147 patients included in the SOAP study, 1,120 (36%) had ARF at some point during their ICU stay. Sixty-day mortality rates were 36% in patients with ARF and 16% in patients without ARF (*P *< 0.01). Oliguric patients and patients treated with renal replacement therapy (RRT) had higher 60-day mortality rates than patients without oliguria or the need for RRT (41% versus 33% and 52% versus 32%, respectively; *P *< 0.01). Independent risk factors for 60-day mortality in the patients with ARF were age, Simplified Acute Physiology Score II (SAPS II), heart failure, liver cirrhosis, medical admission, mean fluid balance, and need for mechanical ventilation. Among patients treated with RRT, length of stay and mortality were lower when RRT was started early in the course of the ICU stay.

**Conclusion:**

In this large European multicenter study, a positive fluid balance was an important factor associated with increased 60-day mortality. Outcome among patients treated with RRT was better when RRT was started early in the course of the ICU stay.

## Introduction

Despite significant improvements in intensive care medicine, the prognosis of acute renal failure (ARF) remains poor, with mortality rates ranging from 40% to 65% [1]. Moreover, ARF continues to influence the prognosis of intensive care survivors even after discharge, decreasing their 3-year survival rate [2]. Several factors may contribute to the high mortality rate of ARF, including the underlying disease [3-6], the circumstances leading to the development of ARF [4-8], the presence of anemia [9], and the severity of illness [4,10]. In addition, therapeutic measures such as mechanical ventilation and the use of vasopressors have been demonstrated to be related to intensive care unit (ICU) mortality in patients with ARF [5,11]. The management of ARF in the ICU patient is very heterogeneous, with little consensus about therapeutic measures such as fluid administration, vasopressors, diuretics, and timing of renal replacement therapy (RRT).

The aim of the present study was to analyze whether fluid management influences mortality in critically ill patients with ARF. To investigate this question, we used the database of the Sepsis Occurrence in Acutely Ill Patients (SOAP) study [12], a large systematic cohort study performed in European ICU patients.

## Materials and methods

### Study design

The SOAP study was a prospective multicenter observational study designed to evaluate the epidemiology of sepsis in European countries and was initiated by a working group of the European Society of Intensive Care Medicine. Details of recruitment, data collection, and management are provided elsewhere [12]. Briefly, all adult patients (>15 years old) admitted to the participating centers between 1 and 15 May 2002 were included (a list of participating countries and centers is given in the Appendix), except patients who stayed in the ICU for less than 24 hours for routine postoperative observation. Since this observational study did not require any deviation from routine medical practice, institutional review board approval was either waived or expedited in participating institutions and informed consent was not required. Patients were followed up until death, hospital discharge, or for 60 days.

### Data collection and management

Data were collected prospectively using preprinted case report forms. Detailed instructions, explaining the aim of the study, instructions for data collection, and definitions for several important items were available for all participants on a dedicated website before starting data collection and throughout the study period. The steering committee processed all queries during data collection. Data were entered centrally by medical personnel.

Data collection on admission included demographic data, comorbid diseases, and admission diagnosis. Considered comorbidities included the presence of insulin-dependent diabetes mellitus, chronic obstructive pulmonary disease, hematological malignancy, solid malignancy, cirrhosis, heart failure class III or IV according to the New York Heart Association definitions, and the presence of HIV infection. Clinical and laboratory data needed to calculate the Simplified Acute Physiology Score II (SAPS II) were reported as the worst value within 24 hours after hospital admission. Serum creatinine level, urine output, and fluid balance were recorded on a daily basis. A daily evaluation of organ function according to the sequential organ failure assessment (SOFA) score was performed, based on the most abnormal value for each of the six organ systems on admission and every 24 hours thereafter.

### Definitions

ARF was defined according to the renal SOFA score as a serum creatinine of greater than 3.5 mg/dL (310 μmol/L) or a urine output of less than 500 mL/day. Separate analyses were made in patients with early- and late-onset ARF, oliguric and non-oliguric patients, and patients treated with or without RRT. In addition, patients with early and late initiation of RRT were compared. For these analyses, early onset of renal failure was defined as ARF occurring within the first 2 days of ICU admission and late onset as ARF occurring more than 2 days after ICU admission. Oliguria was defined as a urine output of less than 500 mL per day. Initiation of RRT was defined as early when started within 2 days of ICU admission and as late when started more than 2 days after ICU admission. Mean fluid balance was calculated as the arithmetic mean of the daily fluid balance during the patient's ICU stay. Fluids taken into account were packed red blood cells, starch, dextran, gelatin, albumin, crystalloids, and tube feeds.

### Statistical analysis

Data were analyzed using the Statistical Package for Social Sciences (SPSS) for Windows, version 11.0 (SPSS Inc., Chicago, IL, USA). The Kolmogorov-Smirnov test was used, and histograms and normal-quantile plots were examined to verify the normality of distribution of continuous variables. Non-parametric measures of comparison were used for variables evaluated as not normally distributed. Difference testing between groups was performed using the two-tailed *t *test, Mann-Whitney *U *test, chi-square test, and Fisher exact test as appropriate. To evaluate the influence of baseline characteristics and fluid balance on 60-day mortality in the patients with ARF, we performed a multivariable Cox regression analysis. Variables considered for the Cox regression analysis included age, gender, comorbid diseases, SAPS II, SOFA score, and mean fluid balance. The Cox regression analysis was repeated separately in patients with early ARF and in those with late ARF. Colinearity between variables was excluded prior to modeling. We examined the goodness of fit of the model with residual plots. Kaplan-Meier survival curves were plotted and compared using a signed log-rank test. The multifactorial analysis of variance with repeated measures procedure was used to compare time courses of fluid balance between groups. Values are given as mean ± standard deviation or median and interquartile range if appropriate. All statistics were two-tailed and a *P *value of less than 0.05 was considered significant.

## Results

### Study population

Of the 3,147 patients enrolled in the SOAP study, 1,120 (36%) developed ARF. The baseline characteristics of patients with and without ARF are summarized in Table 1. ARF patients were significantly older, had higher SAPS II and SOFA scores, and more frequently had sepsis on admission. Even when the renal score was left out, ARF patients had a higher SOFA score on admission (Table 1). Of the 1,120 patients with ARF, 842 (75%) had early-onset ARF (occurring within 2 days of ICU admission) and 278 (25%) had late-onset ARF (occurring more than 2 days after ICU admission).

**Table 1:** Baseline characteristics

Characteristic	All patients n = 3,147	No ARF n = 2,027	ARF n = 1,120	*P *value
Age, years	60.6 ± 17.4	59.3 ± 18.0	62.8 ± 16.1	<0.01
Male gender	1,920 (61.7)	1,250 (62.3)	670 (60.6)	0.34
SAPS II	36.5 ± 17.1	33.4 ± 15.6	42.1 ± 18.3	<0.01
SOFA score on admission	4 (2–8)	4 (1–6)	6 (4–9)	<0.01
SOFA without renal score on admission	4 (1–6)	3 (1–6)	4 (2–7)	<0.001
Vasoactive drugs	1,983 (63.0)	1,034 (51.2)	949 (84.7)	<0.01
Mechanical ventilation	2,025 (64.3)	1,248 (61.8)	777 (69.4)	<0.01
Serum creatinine, mg/dL	1.5 ± 1.5	1.0 ± 0.5	2.2 ± 2.2	<0.01
Comorbid diseases, number (percentage)				
Cancer	415 (13.2)	289 (14.3)	126 (11.3)	0.02
Hematologic cancer	69 (2.2)	39 (1.9)	30 (2.7)	0.17
Chronic obstructive pulmonary disease	340 (10.8)	196 (9.7)	144 (12.9)	<0.01
HIV infection	26 (0.9)	12 (0.6)	14 (1.3)	0.39
Liver cirrhosis	121 (3.8)	73 (3.6)	48 (4.3)	0.34
Heart failure	307 (9.8)	174 (8.6)	133 (11.9)	<0.01
Diabetes	226 (7.2)	121 (6.0)	105 (9.4)	<0.001
Category of admission diagnosis, number (percentage)				
Cardiovascular	949 (32.0)	573 (28.3)	376 (33.6)	0.44
Respiratory	560 (18.9)	341 (18.0)	219 (20.3)	0.61
Neurologic	485 (16.3)	359 (17.7)	126 (11.3)	0.62
Digestive	333 (11.2)	206 (10.9)	127 (11.8)	0.67
Trauma	181 (6.1)	142 (7.0)	39 (3.5)	0.70
Monitoring	247 (8.3)	167 (8.2)	80 (7.1)	0.69
Sepsis syndromes	777 (24.7)	423 (20.9)	354 (31.6)	<0.01
Others	392 (12.5)	239 (11.8)	153 (13.7)	0.66
ICU mortality, number (percentage)	583 (18.5%)	245 (12.1)	338 (30.2)	<0.01
60-day mortality, number (percentage)	722 (23.3%)	327 (16.4)	395 (35.7)	<0.01

Data are expressed as mean ± standard deviation, number (percentage), or median (interquartile range). ARF, acute renal failure; ICU, intensive care unit; SAPS II, Simplified Acute Physiology Score II; SOFA, sequential organ failure assessment.

### Outcome

**Figure 1:**
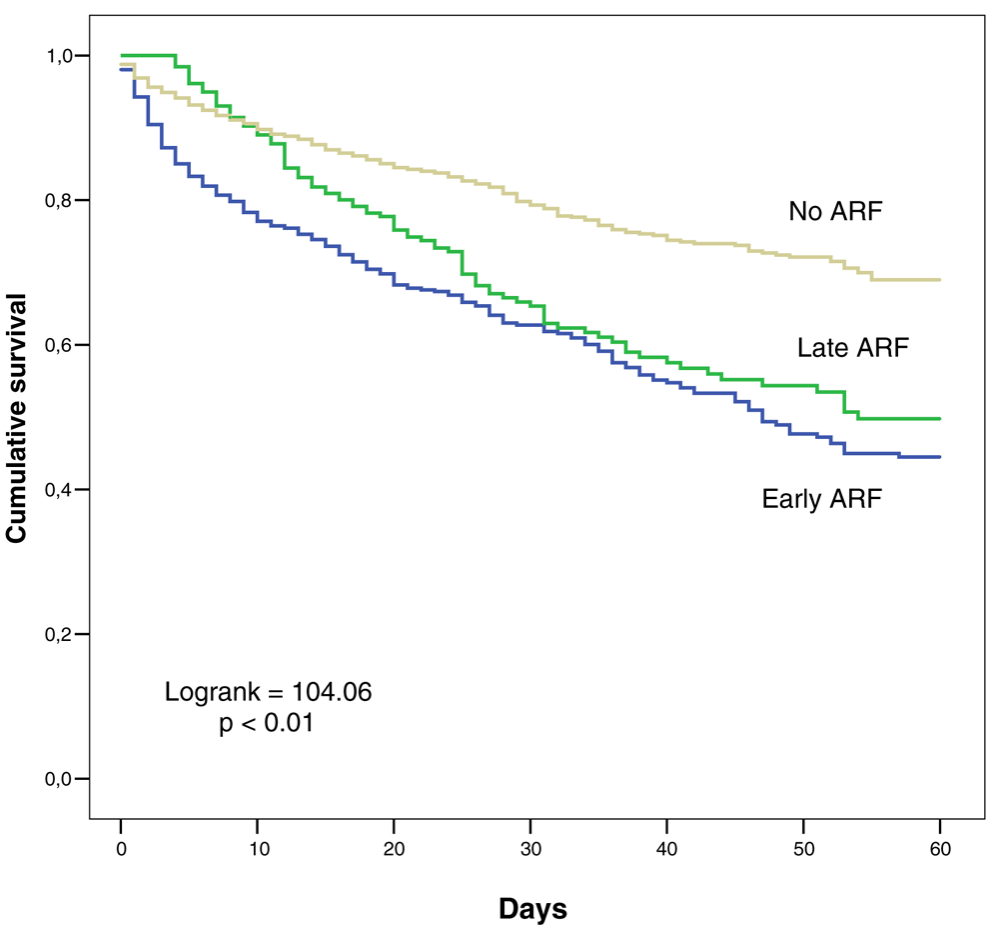
Kaplan-Meier 60-day survival curves in patients without acute renal failure (ARF) and with early- and late-onset ARF.

**Figure 2:**
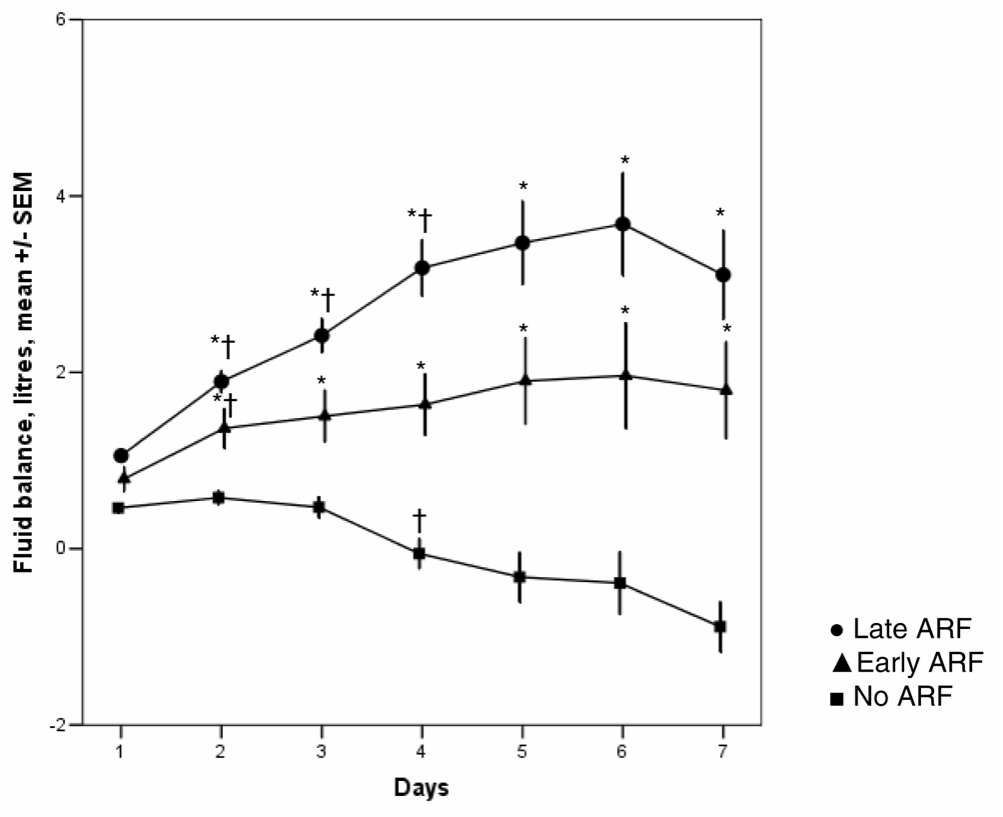
Time course of the daily mean fluid balance during intensive care unit stay in patients without acute renal failure (ARF), with early-onset ARF, and with late-onset ARF. Analysis of variance for repeated measures: *P < 0.05 pairwise compared with each of the two other subgroups; ^†^*P *< 0.05 compared with the previous time point. SEM, standard error of the mean.

Patients with ARF had higher mortality rates than patients without ARF (60-day mortality 35.7% versus 16.4%; *P *< 0.01) (Table 1). Mortality rates in patients with early and late ARF were similar (ICU mortality: 29.2% early, 33.2% late, *P *= 0.21; 60-day mortality: 35.2% early, 37.3% late, *P *= 0.54) (Figure 1). In the Cox regression analysis, seven variables were related to 60-day mortality in the patients with ARF: age, SAPS II, heart failure, liver cirrhosis, medical admission, mean fluid balance, and mechanical ventilation (Table 2). When patients with early- and late-onset ARF were analyzed separately, mean fluid balance was retained as an independent predictor of mortality only in the patients with early ARF. Mean fluid balance was significantly more positive in patients with early and late ARF than in patients without ARF throughout the first 7 days of the ICU stay (Figure 2). In all ARF groups, mean fluid balance was more positive among non-survivors than among survivors (Table 3). To analyze further the determinants of mortality and fluid balance in patients with ARF, we divided the patients into two groups according to urine output and treatment with RRT. In oliguric patients and in patients treated with RRT, mean fluid balance was significantly more positive than in non-oliguric and non-RRT patients, respectively, and mortality rates were significantly higher (Table 4). However, oliguric patients had shorter ICU and hospital stays than non-oliguric patients, whereas patients treated with RRT had longer ICU and hospital stays than non-RRT-treated patients. To analyze the influence of the time of initiation of RRT on outcome, we divided the RRT group into an early and a late RRT group, according to the time elapsed between ICU admission and the start of RRT. As shown in Table 5, patients in the early RRT group were more severely ill on ICU admission, as reflected by higher SAPS II and SOFA scores. Despite this greater severity of illness, length of stay was shorter and mortality was lower in the group in which RRT was started early in the course of the ICU stay (Table 5).

**Table 2:** Hazard ratios: results of multivariate Cox regression analysis for 60-day mortality in critically ill patients with acute renal failure

Characteristic	Hazard ratio	95% CI	*P *value
Age	1.02	1.01–1.03	<0.001
SAPS II (per point)	1.03	1.02–1.04	<0.001
Heart failure	1.38	1.05–1.81	0.02
Medical admission	1.68	1.35–2.08	<0.001
Mean fluid balance, L/24 hours	1.21	1.13–1.28	<0.001
Mechanical ventilation	1.55	1.14–2.11	<0.001
Liver cirrhosis	2.73	1.88–3.95	<0.001

CI, confidence interval; SAPS II, Simplified Acute Physiology Score II.

**Table 3:** Mean daily fluid balance among 60-day survivors and non-survivors with acute renal failure (ARF), stratified by time of onset

Mean fluid balance, L/24 hours	Survivors	Non-survivors	*P *value
ARF	0.15 ± 1.06	0.98 ± 1.50	<0.001
Early ARF (occurring within 2 days of ICU admission)	0.14 ± 1.05	1.19 ± 1.48	<0.001
Late ARF (occurring more than 2 days after ICU admission)	0.11 ± 1.03	0.39 ± 1.40	0.06

ICU, intensive care unit.

**Table 4:** Mean daily fluid balances and outcome among patients with acute renal failure, stratified by urine output and treatment

Characteristic	Non-oliguric n = 572	Oliguric n = 548	*P *value	No RRT n = 842	RRT n = 278	*P *value
Mean fluid balance, L/24 hours	0.27 ± 1.23	0.62 ± 1.33	<0.01	0.39 ± 1.21	0.60 ± 1.50	<0.01
ICU mortality	157 (27.4)	181 (33.0)	0.04	214 (25.4)	124 (44.6)	<0.01
60-day mortality	181 (32.1)	214 (39.6)	0.01	259 (31.2)	136 (49.5)	<0.01
ICU stay	4.5 (2.0–11.1)	3 (1.4–8.6)	<0.01	2.9 (1.6–6.9)	8.4 (3.0–19.4)	<0.01
Hospital stay	12.7 (5.5–21.0)	10.3 (2.3–22.2)	<0.01	10.8 (3.8–24.1)	16 (6.8–34.9)	<0.01

Data are expressed as mean ± standard deviation, median (interquartile range), or number (percentage). ICU, intensive care unit; RRT, renal replacement therapy.

**Table 5:** Characteristics of patients with acute renal failure, stratified by time of initiation of renal replacement therapy (RRT)

Characteristic	Early RRT n = 213	Late RRT n = 65	*P *value
Age	62.3 ± 15.5	64.6 ± 15.0	0.30
Male gender	126 (59.4)	44 (68.8)	0.18
SAPS II	49.7 ± 17.5	45.3 ± 18	0.04
SOFA score	9.2 ± 4.1	8.2 ± 3.5	0.04
Mechanical ventilation	166 (77.9)	61 (93.8)	<0.01
Type of admission			
Medical	87 (40.8)	38 (58.5)	0.01
Surgical	126 (59.2)	27 (41.5)	0.01
Urine output, L/24 hours	0.18 (0.03–0.50)	0.47 (0.09–1.74)	<0.001
Creatinine, mg/dL	3.99 (2.57–6.17)	3.29 (2.10–5.00)	0.06
ICU stay, days	6.1 (2.5–14.8)	12.2 (8.0–26.5)	<0.001
Hospital stay, days	25.0 (8.0–46.0)	27.0 (17.0–45.0)	0.10
ICU mortality, number (percentage)	84 (39.4)	40 (61.5)	<0.01
60-day mortality, number (percentage)	94 (44.8)	42 (64.6)	<0.01

Data represent mean ± standard deviation, number (percentage), or median (interquartile range). ICU, intensive care unit; SAPS II, Simplified Acute Physiology Score II; SOFA, sequential organ failure assessment.

## Discussion

We studied the occurrence of ARF among patients admitted to European ICUs, except those admitted for routine postoperative surveillance, and analyzed the influence of patient characteristics and fluid balance on outcome. Patients developing ARF were older, were more severely ill, and more frequently had sepsis on admission to the ICU. Crude mortality rates were higher in patients with ARF than in those without ARF, particularly in patients with oliguria and those treated with RRT. Cox regression showed that age, SAPS II, heart failure, liver cirrhosis, medical admission, mean fluid balance, and mechanical ventilation were independently related to 60-day mortality.

A relation between a positive fluid balance and an unfavorable ICU outcome has been described before in general ICU populations. Mitchell and colleagues [13] demonstrated a decrease in ventilator and ICU days in patients treated with fluid restriction and increased diuresis compared with a wedge pressure-guided fluid protocol. Upadya and colleagues [14] demonstrated that a negative fluid balance was independently associated with weaning success in mechanically ventilated patients. In an earlier analysis of the SOAP database, Sakr and colleagues [15] demonstrated that mean fluid balance was an independent determinant of ICU outcome in patients with acute lung injury/adult respiratory distress syndrome. In patients with sepsis, the relation between a positive fluid balance and a negative outcome has also been described. Alsous and colleagues [16] demonstrated an increased mortality risk in patients failing to achieve a negative fluid balance within the first 3 days of treatment (relative risk 5.0, 95% confidence interval 2.3 to 10.9). However, the relation between fluid balance and outcome of patients with ARF has not been extensively studied. Van Biesen and colleagues [17] demonstrated that septic patients developing ARF had a higher colloid fluid loading, a higher central venous pressure, and a worse respiratory function than septic patients without ARF.

In this observational study, it was not possible to determine whether the positive fluid balance found in ARF patients was the cause or the result of a greater severity of illness, especially as resuscitation protocols were not standardized and there is considerable debate as to the optimal approach to fluid management in critically ill patients with ARF. Indeed, in a recent review, Mehta and colleagues [18] concluded that there are no established guidelines for the modulation of fluid balance by means of diuretics or RRT in patients with organ dysfunction. Although diuretics can influence fluid balance, there is no evidence that their use can alter outcome in ARF [19], and the evidence for a beneficial effect of RRT on outcome in patients with multiple organ failure is also debated [20-22]. The findings of the present study should stimulate further studies to investigate the role of fluid balance on prognosis in these patients.

Mortality rates in our study were similar in early- and late-onset ARF, which is in contrast to the findings of Brivet and colleagues [23]. In a prospective multicenter study of 360 ICU patients with ARF, these authors found a higher hospital mortality rate in patients with delayed-onset ARF compared with initial ARF (71% versus 50%; *P *< 0.001), with an odds ratio for mortality of 2.97 (95% confidence interval 1.72 to 5.13). Brivet and colleagues reported no difference in disease severity between patients with initial and delayed-onset ARF. Patients with delayed-onset ARF even had a lower serum creatinine level (346 ± 7 versus 550 ± 19 μmol/L; *P *< 0.001). As patients with delayed-onset ARF did not have worse baseline characteristics, one could argue that therapeutic factors or complications may have contributed to the worse outcome in these patients.

Another interesting finding in the present study is that outcome among patients treated with RRT was better when RRT was started early in the course of the ICU stay. Although in the present study we studied initiation of RRT in relation to ICU admission (rather than to the onset of ARF as in most other studies), our results do agree with the findings of several retrospective studies that suggest that early initiation of RRT may be beneficial in ARF patients [24-27]. The results of a recent prospective multicenter observational study also support our findings, with late RRT (defined as being initiated more than 5 days after ICU admission) being associated with greater crude and covariate-adjusted mortality compared with early (within 2 days) or delayed (2 to 5 days) initiation of RRT [28]. However, a prospective randomized study in a mixed ICU population found no difference in survival between early (on average within 7 hours of development of ARF) and late (on average 42 hours after development of ARF) initiation of RRT [29].

## Conclusion

This large multicenter European observational study confirms the high mortality rate of ARF in critically ill patients. It also confirms the finding that oliguric patients and patients treated with RRT have a worse outcome. In addition, it uncovers the importance of a positive fluid balance as a strong outcome predictor in critically ill patients with ARF.

## Key messages

• In this large multicenter European observational study in critically ill patients, 60-day mortality rate among patients with acute renal failure (ARF) was more than twice as high as among other patients (35.7% versus 16.4%; *P *< 0.01).

• In patients with ARF, mean daily fluid balance was significantly more positive among non-survivors than among survivors (0.98 ± 1.5 versus 0.15 ± 1.06 L/24 hours; *P *< 0.001).

• Among oliguric patients and patients treated with renal replacement therapy (RRT), mean daily fluid balance was significantly more positive (0.62 ± 1.33 versus 0.27 ± 1.23 L/24 hours; *P *< 0.01, and 0.60 ± 1.5 versus 0.39 ± 1.21 L/24 hours; *P *< 0.01) and 60-day mortality rates were significantly higher (39.6% versus 32.1%; *P *< 0.01, and 49.5% versus 31.2%; *P *< 0.01).

• Among patients in whom treatment with RRT was started early in the course of ICU admission, median length of ICU stay was significantly shorter (6.1 versus 12.2 days; *P *< 0.001) and 60-day mortality rate was significantly lower (44.8% versus 64.6%; *P *< 0.01).

## Abbreviations

ARF = acute renal failure; ICU = intensive care unit; RRT = renal replacement therapy; SAPS II = Simplified Acute Physiology Score II; SOAP = Sepsis Occurrence in Acutely Ill Patients; SOFA = sequential organ failure assessment.

## Competing interests

The authors declare that they have no competing interests.

## Authors' contributions

DP and J-LV designed the study. ACdP wrote the manuscript drafts. YS was responsible for the analysis of the data. All authors participated in the acquisition of the data and contributed in the writing and critical appraisal of the manuscript. All authors read and approved the final manuscript.

## Supplementary Material

Additional file 1 The additional file consists of a list of participants to the Sepsis Occurrence in Acutely Ill Patients (SOAP) study in alphabetical order. Click here for file
